# Ranking Series of Cancer-Related Gene Expression Data by Means of the Superposing Significant Interaction Rules Method

**DOI:** 10.3390/biom10091293

**Published:** 2020-09-08

**Authors:** Emili Besalú, Jesus Vicente De Julián-Ortiz

**Affiliations:** 1 Institut de Química Computacional i Catàlisi (IQCC) and Departament de Química, Universitat de Girona, 17003 Girona, Spain; 2 Molecular Topology and Drug Design Research Unit, Departament de Química Física, Facultat de Farmàcia, Universitat de València, 46100 Burjassot, Spain; jejuor@uv.es

**Keywords:** SSIR method, multilevel fingerprints, gene expressions, cancer, prostate cancer, leukemia, ranking

## Abstract

The Superposing Significant Interaction Rules (SSIR) method is a combinatorial procedure that deals with symbolic descriptors of samples. It is able to rank the series of samples when those items are classified into two classes. The method selects preferential descriptors and, with them, generates rules that make up the rank by means of a simple voting procedure. Here, two application examples are provided. In both cases, binary or multilevel strings encoding gene expressions are considered as descriptors. It is shown how the SSIR procedure is useful for ranking the series of patient transcription data to diagnose two types of cancer (leukemia and prostate cancer) obtaining Area Under Receiver Operating Characteristic (AU-ROC) values of 0.95 (leukemia prediction) and 0.80–0.90 (prostate). The preferential selected descriptors here are specific gene expressions, and this is potentially useful to point to possible key genes.

## 1. Introduction

Cancer is one of the leading causes of death worldwide, representing an estimate of 9.6 million deaths in 2018 [1].

In light of current research, a decrease in cancer frequency overall is basically reliant on diminishing exposure to known cancer-causing agents [2]. In any case, the currently available research on different types of cancer, including prostate cancer and leukemias, do not permit a clear proportion of these malignancies to be ascribed to specific exogenous factors. Genomics and other technologies are critical to future etiology investigations and to fine-tuning improved procedures for early diagnosis [2].

Leukemias are a heterogeneous group of more than 30 lymphoid and myeloid malignancies with differing etiologies, treatment pathways, and results [3]. In 2018, there was an estimate of 4.37 × 10^5^ new cases of leukemia around the world, and leukemia was the fifteenth most prevalent type of cancer, representing 2.4% of all new cancer cases [3]. In some regions, leukemia can be the most common type of malignant tumor, probably associated with the presence of contamination by agrochemicals [4].

Leukemias pose diagnostic problems. Since numerous countries do not yet have high-quality, representative malignancy registration frameworks, looking at worldwide variety and trends after some time is challenging for any type of cancer. For leukemias, the circumstance is exacerbated by diagnostic difficulties related to recognizing distinctive leukemia subtypes alongside inconsistent implementation of the WHO classification [3,5]. Moreover, even in countries with good cancer registry systems, there is an absence of consistency in policies applied to progressions and transformations. For instance, the United States Surveillance, Epidemiology, and End Results (SEER) program has different guidelines in comparison to the European Network of Cancer Registries [6]. In low-income countries, where mortality and morbidity from infections and nourishing conditions are regularly high, diagnosing leukemia presents extra difficulties. The symptoms of many types of leukemia are fundamentally the same as those of infectious and parasitic diseases. On the other hand, both experience and diagnostic methods to distinguish leukemia from background infections are often poor.

Genomics is utilized for diagnosis, classification, and leukemia prognosis. DNA sequencing and next-generation sequencing array-based platforms provide the best genomic resolution, demonstrating the complexity of numerous leukemia subtypes [7] beyond chromosomal translocation and aberrant fusion proteins.

Prostate cancer is currently the second most common non-cutaneous cancer in men around the world and the fourth most common cancer, with an estimated 1.28 million new cases occurring in 2018, representing 13.5% of new cancer cases in men [1]. Their mortality is not that significant, representing 3.6 × 10^5^ deaths (6.7% of cancer deaths in men) in 2018 [2]. However, it is highly heritable [8]. The introduction of prostate-specific antigen (PSA) testing as a diagnostic test has largely been the cause of good prognosis for this type of cancer [9]. The most common prostate cancer subtype is acinar adenocarcinoma, which accounts for more than 99% [10].

A high risk of prostate cancer has been associated with mutations in the BRCA2 and HOXB13 genes [11]. Genomic associations at 8q24 [12], DNA mismatch repair gene mutations, as well as regulation of the downstream gene MYC or regulation by long noncoding RNAs has been related to higher morbidity risk in African-descent men [13].

Biomarkers that predict the probability of aggressive disease include the TMPRSS2–ERG gene fusion [14], Ki-67 expression [15], and biomarkers involved in androgen metabolism [16]. Multigenic genomic classifiers have been recognized that assess the forecast or the aggressiveness of the prostate tumor [17]. Cell lines of acute myeloid leukemia and prostate cancer express the full-length androgen receptor [18].

Classification of DNA microarray data is a hot topic in the field of bioinformatics, since it is an effective tool for the diagnosis of diseases in patients. Many approaches have been used in feature selection methods to analyze microarray datasets for the diagnosis and stratification of patients in these types of cancers [19], and a review is not attempted here, but it can be seen in [20]. Typically, two main methodologies are utilized to examine microarray gene expression datasets: supervised learning (class prediction) and unsupervised learning (class discovery). [21] Supervised learning algorithms group tumors into known classes and create transcription profiles of defined groups, using different technologies. An example is the k-nearest neighbor algorithm, that defines a distance and classifies features with respect to subset clusters of data. Unsupervised learning groups sample in clusters based on the general similarity of their gene expression profiles without prior information on particular relationships [22]. Microarray data have two specific qualities that differentiate most data sets used in programmed classification tasks: high dimensionality and a small number of instances. This causes a considerable amount of machine learning strategy struggles in completing the grouping task in a manner that generalizes appropriately, for the main part, because of data overfitting issues or curse of dimensionality. Hence, many of the techniques utilized depend on simple linear models or on nonlinear models explicitly designed to alleviate the issues of overfitting. The problem of simple techniques is that they cannot afford a good model for too complex data.

Golub et al. [23] used, for this purpose, signal-to-noise feature selection and statistical analysis. A conclusion of the study was that only a portion of these large data in a DNA microarray was relevant for solving the classification problem. The dataset used in the present article has also been taken from this reference. More powerful methods are now available for the same purpose that had been applied to the same dataset [24,25,26].

Among the last papers that used the Golub dataset, the following stand out. Lin el al [24] have proposed an Interaction Gain-Recursive Feature Elimination technique that evaluates the relevance of a feature by an iterative method which combines the relevance between feature and class labels and the interaction among features. They also compare the performance of their method with other previously described methods, and the new method outperforms them. Alanni el al. [25] have presented a new method for selection of relevant genes. This is an evolutionary programming algorithm that improves the previous technology and uses a support vector machine to evaluate its performance. Eraslan et al. [26] have reviewed the application of deep learning techniques in the same problem, including diverse types of neural networks. All the algorithms included in these studies have the drawback of requiring intensive calculations to obtain the parameters of the respective models. For this reason, we looked for a method able to retain the complexity of the data without the price of long CPU time.

On the other side and totally independent of the previous descriptions, the Superposition of Significant Interaction Rules (SSIR) method has already been described in several places, demonstrating versatility and good performance, for instance, modelling biological activities [27,28,29]; showing the link between the method and Design of Experiments (DoE) techniques [30]; or in this journal, preprocessing chromatographic data for ulterior analysis and samples classification [31]. Here, it is presented for the first time as an application that ranks dichotomized sets of samples that are described by binary or multilevel descriptors (fingerprint-like) that encode gene expressions. It is shown how fingerprint encoding is related to the generation of rules and how, in turn, these rules can be statistically quantified using a significant *p*-value. The most significant rules are selected and constitute a consensus-like voting system rule set, which is used to rank the series of analyzed items or samples. This allows for the classification or sorting (ranking) of gene expressions among individuals.

As will be shown below, a rule consists of the specification of one or more variables and the respective selected levels. Each rule definition is compatible with (embraces or condenses) a set of samples, classified a priori as belonging to a binary class (e.g., active or non-active, and expressing or not expressing a gene). Counting the number of condensed samples that are of either class provides a probability associated with the rule. This probability leads to the quantification of a statistical significance. The calculation of the level of significance associated with the rule is performed using the hypergeometric experiment [32].

Here, the application to the Structure–activity Relationship (SAR) field of a series of samples described by multilevel fingerprints encoding gene expressions is explored for the first time. This study constitutes a demonstration that the algorithm is efficient for this type of data and that a simple and fast procedure is useful for patient classificatory purposes. For leukemia, AU-ROC values of 0.95 for prediction were obtained and good results from a randomization test were also obtained. Concerning prostate cancer, the AU-ROC values are 0.80–0.90 for leave-one-out (L1O) calculations.

## 2. Materials and Methods

### 2.1. Hypergeometric Probability Experiment: The Urn Experiment

Consider the experiment consisting of randomly drawn *c* marbles without replacement of a set of *a* (where *b* of them are “of interest” or “active” and *a*, *b* are of “non-interest” or “inactive”), such that, after selection, *d* of the collected ones appear to be of interest (and the remaining *c*–*d* are not of interest). If all the marbles are assumed to have a priori the same probability to be extracted, the probability associated with the described process follows the hypergeometric probability distribution [32]:

(1) $$P\left(d,c;b,a\right)=\frac{\left(\begin{array}{c} b\\ d \end{array}\right)\left(\begin{array}{c} a-b\\ c-d \end{array}\right)}{\left(\begin{array}{c} a\\ c \end{array}\right)}\;\mathrm{with}\;d\leq \;c\leq \;a\;\mathrm{and}\;d\leq \;b\leq \;a.$$

where the minimum allowed value for *d* is max(0, *c* + *b* − *a*), whereas the maximum is min(*b*, *c*). The mean and the variance of the hypergeometrical random variable *d* is respectively the following:

(2) $$\mu =c\frac{b}{a}\;\mathrm{and}\;\sigma^{2}=\frac{bc\left(a-b\right)\left(a-c\right)}{a^{2}\left(a-1\right)}$$

The SSIR procedure relies on previous statistical concepts. There is a parallel between the classical urn statistical experiment and the underlying SSIR concepts:

(a) The classic urn experiment consists of randomly removing a number of marbles from a container. The counterpart in the SSIR method is the generation of a *rule* (see below) which implies the choice of several samples selected from the pool of available ones. In the applications shown below, the samples are individuals which are described by gene expressions.
(b) The classic urn procedure assumes that the selection of marbles is totally random and that each marble has the same probability of being chosen. As will be explained below, the SSIR procedure generates systematic and deterministic rules, each of which selects a subset of individuals. It is assumed that this selection can be considered a priori random because, in principle, each sample can be paired with any other by forming subsets.
(c) The marbles exhibit one of two possible colors, in the same way that each individual has been previously labelled in a dichotomic fashion, for instance, presenting cancer or not.
(d) In the urn experiment, it is known in advance how many marbles the urn contains and how many of them are of either color. Equivalently, it is known in advance how many individuals are investigated (i.e., constitute the training sample set) and which is its binary classification condition (e.g., has cancer or not, presents a cancer type in its form A or form B, etc.).
(e) After a random extraction of a certain number of marbles, the hypergeometric formula gives the probability of extracting the particular number of marbles of a predefined color among the selected subset. By simple addition, the formulation gives the probability of extracting at least a certain number of marbles of a specific color. This last added probability constitutes a *p*-value (see below). Similarly, a rule acts as an “extractor” and the probability of extracting the particular number of individuals of a predefined binary case (disease condition) can be also evaluated with the same formulation as in the former case. Consequently, the hypergeometric formulation is used to associate probabilities and a *p*-value to each rule or extraction.

The SSIR procedure is based on the systematic generation of rules and their statistical evaluation. As explained above, a sample set of *a* individuals was previously dichotomized according to an experimental property or disease condition: *b* members are of one kind and the remaining *a*-*b* individuals conform the complementary kind. Then, the SSIR method systematically constructs selector rules of subgroups of *c* individuals among the set of the available *a*. After each selection, i.e., after each rule, the number of cases of a certain kind are counted, *d*, and a level of significance, or *p*-value, is attached to the considered rule. Here, the null hypothesis is that a rule cannot select a number of individuals beyond the expected class mean. Hence, the *p*-value is the sum of probabilities in which the rule covers the actual number of samples of a certain kind, *d*, or even more (we denote this condition as *d*+):

(3) $$p\left(d+,c;b,a\right)=p\left(d:\min\left(b,c\right),c;b,a\right)=\sum_{i=d}^{\min\left(b,c\right)}P\left(i,c;b,a\right)=1-\sum_{i=\max\left(0,c+b-a\right)}^{d-1}P\left(i,c;b,a\right)$$

The rules can be ordered by preferences, the most relevant ones being those that present the minimum value of probability *p*. In fact, the actual version of the SSIR code only takes into account the set of all the rules that have a *p*-value lesser than or equal to a predefined cutoff *p_c_*. This set of rules will act as an expert system that will grant votes to the samples. The set of votes collected per sample will define the overall items or individuals ranking.

### 2.2. Rules

Let us consider a samples library where each individual is described by means of strings encoding *n* characters or gene expressions, for instance, *C*_1_*C*_2_*C*_3_…*C_n_*. Each character *C_i_* presents a set of allowed possibilities (e.g., levels of gene expression). For example, at position *i*, there are a total of *m* levels available for the character *C_i_* = {*L*_1_, *L*_2_, …; *L_m_*}. Formally, the entire virtual library is expanded combinatorically by the Cartesian product *C*_1_ × *C*_2_ × … × *C_n_*. In general, not all combinations of levels are feasible.

The generation of SSIR rules is carried out in a systematic and combinatorial mode (despite non exhaustive procedures being also envisaged). In References [27,29], the reader can find additional details of the algorithm and its rationale. An SSIR rule consists of the specification (by inclusion or exclusion) of some levels for sites. For instance, considering a fingerprint of 4 characters in length, all individuals presenting the particular level *A* in the first encoded gene are expressed by the rule *AXXX*. Here, the symbol “*X*” represents the “all-levels” wildcard. This rule is called of order 1 because only one gene level is specified, as is also the case for rule *XBXX*. The potential full database is expressed as *XXXX*, which is the unique rule of order 0. As another example, the subset of individuals bearing the first allowed level in the first character and *simultaneously* the second level at the third position is expressed by the rule *AXBX*, constituting a rule of order 2. SSIR algorithm is potentially able to generate all rules of order *k* = 1, …, *n*. However, due to the combinatorial explosion, in some cases, only rules up to orders 3 or 4 can be handled comprehensively. Despite this, experience indicates that higher-order rules are normally not useful at all, as is the general case in DoE theory [30] when considering combinations of three or more factor levels. Note that each defined rule will act as an extractor agent that performs a pseudo-random extraction of individuals (see the urn experiment parallelism above).

The above rules are attached to the concept of “presence of a gene level”. The number of rules increases if the concept of “non presence of a specific gene level” is also considered. Taken from the set theory, the negation of a certain level is denoted by a bar. In the example above, the rule $\bar{A}XXX$ expresses the set of individuals that do not show level *A* in the first gene. The combination of two complementary (nonexclusive) rules can lead to selection of other specific subsets or individuals. For example, the rule $\bar{A}\bar{B}XX=\bar{A}XXX\cap X\bar{B}XX$ represents the sublibrary of individuals that do not have level *A* in the first character and that *simultaneously* do not have level B at the second expressed gene. More information on rule algebra can be found in Reference [27].

From a computational point of view, the systematic generation of extractors or rules is performed nesting three combinatorial entities. The first one explicitly generates combinations among *k* fingerprint positions in order to establish the rule order and the second one generates variations with repetition among the levels attached to the previously selected string positions. Eventually, a third one generates 2*^k^* binary numbers, constituting a flag for encoding of all possible combinations of negations or non-negations. See Reference [29] for exhaustive information on how to implement the appropriate algorithms.

### 2.3. Rules with Positive or Negative Votes

Consider the example of an available library consisting of *a* = 100 persons where only *b* = 20 of them developed a certain disease. Suppose also that a total of *c* = 10 of these persons conform to the rule *AXBX* (that is, they are the unique individuals that present levels *A* and *B* in the first and third gene encodings, simultaneously). This rule is then said to condense or represent these individuals. As stated above, the rule produces a virtual a priori random extraction of 10 elements and, at the same time, constitutes a selection of common underlying characteristics. Of these 10 extracted persons, we will now assume that *d* = 9 of them developed the disease. The considered rule seems to be a good one because the diseases proportion *b*/*a* = 20/100 of the whole library is less than the proportion *d*/*c* = 9/10 for the selected persons (related to the above null hypothesis, that is the same as saying that *d* > µ = *cb*/*a*). Thus, the rule constitutes a sort of “disease enrichment” operator. The probabilistic calculation informs about the statistical significance or importance of the rule. From Equations (1) and (3), the probability of collecting 9 positive cases is *P* (9, 10; 20, 100) = 7.76·10^−7^ and the probability of collecting 9 or more positives (when selecting 10 at random) is *p*(9+, 10; 20, 100) = *P* (9, 10; 20, 100) + *P* (10, 10; 20, 100) = 7.87·10^−7^. If a cutoff *p*-value of, for instance, *p_c_* = 10^−5^ is set up a priori, the rule *AXBX* is to be considered a good candidate to enter into the disease classifier model or pool of voting agents, since it collects a set of gene levels associated with an increase of the ratio of positive cases.

In this example, the rule *AXBX* is assigned a positive vote or score. However, there are also rules entering into the model which can be attached to negative votes. For instance, if the rule *BCXX* condenses *c* = 30 persons from the library (20 out of 100 are positives) and only two of them developed the disease (*d* = 2), its level of significance is the one found to collect two or more positives: *p* (2+, 30; 20, 100) = 0.996. This large value indicates that the *BCXX* rule should apparently be avoided because it mainly collects samples of no interest. Fortunately, this still constitutes helpful information. This is so because the sum of the entire range of probabilities *P* adds the following unit:

*p*(max(0, *c* + *b* − *a*):min(*c*, *b*), *c*; *b*, *a*) = 1 (4)

Consequently,

*p*(*d*−, *c*; *b*, *a*) + *p*((*d* + 1)+, *c*; *b*, *a*) = 1 (5)

where *d*− stands for “*d* active samples or less”. Then,

*p*(*d*−, *c*; *b*, *a*) = 1 − *p*((*d* + 1) +, *c*; *b*, *a*) = 1 − *p*(*d*+, *c*; *b*, *a*) + *P*(*d*, *c*; *b*, *a*) (6)

which indicates that the probability of selecting *d* or less cases of disease can be a small number if *p* (*d*+, *c*; *b*, *a*) is large enough. In our example, *p* (2−, 30; 20, 100) = 0.023. For many purposes, this will be a significant result. As a consequence, the rule *BCXX* could be also included in the model but attached to a negative penalty vote as it is expected that the cases having this variable pattern will probably belong to the opposite side of the wanted effects. 

References [19,21] show how a rule presenting a negative vote defines a dual rule that bears a positive vote. The respective significances of both rules appear in the next equation:

*p*(*d*−, *c*; *b*, *a*) = *p*([*b* − *d*]+, *a* − *c*; *b*, *a*). (7)

Both rules are related by a negation operator, and in terms of set theory, each rule defines a subset that is complementary of the other. Hence, continuing with the *BCXX* rule example, due to (7), the significant term *p* (2−, 30; 20, 100) is equal to *p* (18+, 70; 20, 100). Obviously, it is of the same difficulty (in fact, it is equivalent) to select two or less active samples in a set of 30 taken from the library as to pick up 18 or more of the active items when performing an extraction of 70. This is so because, in our formulation, the meaning of the two values of the dichotomized property variable can be reversed. The concept of reversal is related to the complementary or negative rule of *BCXX*: its opposite event is the total negation $\bar{BCXX}=\bar{B}XXX\cup X\bar{C}XX$, i.e., the rule which avoids simultaneous combination of levels *B* and *C* at string positions 1 and 2, respectively. Therefore, it is equivalent to speak in terms of a rule having a negative vote and to consider the negated rule but with a positive one.

### 2.4. Classification or Ranking Models

Generating a classification or a ranking model involves selecting a set of significant rules and assigning votes to each individual. After selecting the significant rules, each case will cumulate the votes of all the rules that condense it. This step constitutes a superposition of rules and provides a sum of votes or scores to each individual, and these values are used to rank the case samples. The ultimate goal is to apply this voting scheme also to new individuals that are not present during the training process and to rank them properly. The originated ranking is expected to establish a preference sorting that increases the chances of early targeting of new samples of interest.

The procedure just mentioned constitutes a simple fitting or training method (together with an eventual external testing). Distinct test or validation schemes can be easily implemented in SSIR [27,29]. Here, leave-one-out (L1O) or randomization test results will be presented below.

### 2.5. A Toy Example

**Figure 1:**
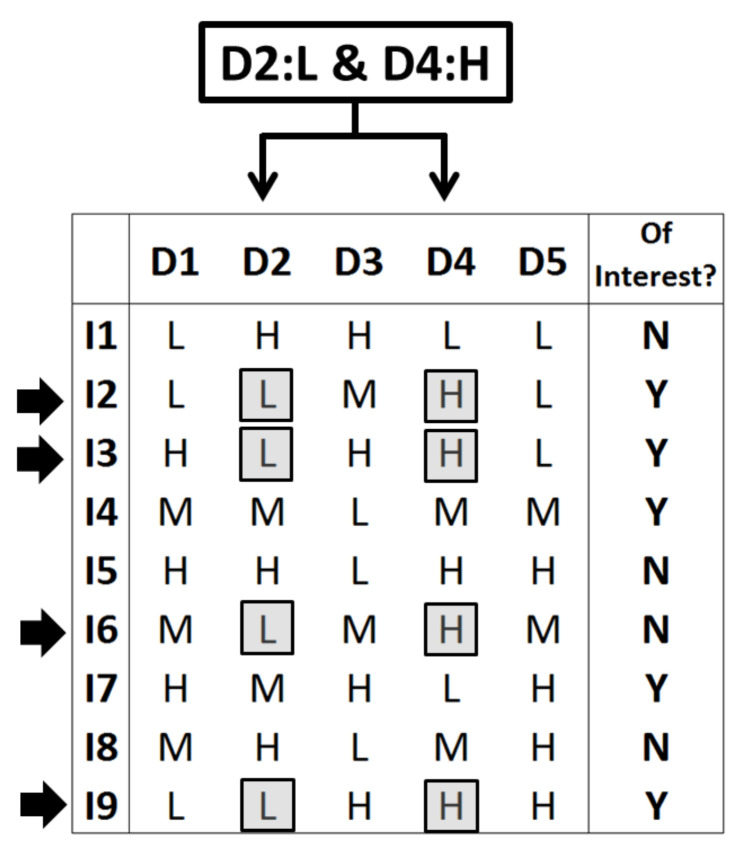
How a rule of order 2 selects over a set of 9 individuals represented by 5 descriptors: See the text for details.

Before presenting the results, for illustrative purposes, this subsection presents a toy example in order to show what a rule is, how it acts over a set of individuals, and how it determines a selection of a subset of individuals. Figure 1 represents the needed data: Across a set of 9 individuals (I1–I9), each one is declared in the last binary column as being or not of “interest” (suffering cancer or not or presenting subtype 1 or 2 of a certain cancer type or any other binary variable). Each individual is represented here by five descriptors (columns D1–D5), which can present one of three levels (low, medium, or high or, equivalently, L, M, and H). Each row of descriptors can be understood here as a string collecting five gene expressions.

As said in the text, the SSIR procedure operates by generating rules. Figure 1 considers a particular rule (of order 2) consisting of the exhibition of the low level in descriptor D2 and, simultaneously, the high level in descriptor D4 (rule “D2: L and D4: H” or, equivalently and according to the above notation, rule *XLXHX*). This rule implies the selection of all individuals that fulfil the specified double condition. In this case, there are 4 individuals (I2, I3, I6, and I9) that satisfy the rule. Therefore, this rule selects this specific subset of individuals.

Qualitatively, the rule seems to be relevant because it embraces 3 individuals of interest (I2, I3, and I9) and this proportion of 3/4 inside the subset is superior to the proportion found for the whole set of individuals: 5 of interest out of 9, or 5/9. This simple information can sometimes suffice to maintain the rule (i.e., include it in the consensus pool) and to add votes to the implied descriptors D2 and D4 (i.e., setting preferences among descriptors, pointing to the presumably key ones to discern inside the binary property). In general, SSIR computes a *p*-value in order to evaluate the efficiency of a rule (i.e., to quantify the difficulty to pick up a certain number of individuals of interest when a random subset is selected). In this particular toy example, the values appearing in Equations (1) and (3) are *a* = 9 individuals in total, with *b* = 5 of them being of interest; the considered rule selects *c* = 4 individuals, with *d* = 3 of them being of interest. Hence, the probability of this event, according to Equation (1), is *P*(3, 4; 5, 9) = 20/63 and the probability to find 4 individuals of interest when selecting 4 is *P*(4, 4; 5, 9) = 5/126. By simple addition, Equation (3) provides the probability to find three or more individuals of interest in this set when four are randomly selected: this is the *p*-value of *p*(3+, 4; 5, 9) = *P*(3, 4; 5, 9) + *P*(4, 4; 5, 9) = 45/126 or a 35.7% chance. Usually, this is not a small enough *p*-value for real purposes, but it has been obtained here due to simplicity of the example (only a total of 9 individuals have been considered).

The SSIR procedure generates all possible rules or a certain order (combining descriptors and levels) and only keeps those rules bearing a small enough *p*-value. This set of selected rules can be used for two purposes. First, this process of rule selection implies an internal ranking of preferred descriptors (e.g., specific gene expressions): it is assumed that the most used descriptors appearing in the selected rules are more related to the internal law that governs the set dichotomization. For this reason, the SSIR procedure can also act as a variable selector. [31] On the other hand, each individual (either from the training set or from a newly external set) can be faced against each selected rule. Each time the individual matches one of these rules (i.e., in the same way that the 4 above individuals in Figure 1 did), it receives a vote. It is assumed that the more votes an individual receives, the greater the probabilities to belong to the interest subgroup of the dichotomized property. This feature establishes a ranking for the individuals.

### 2.6. Datasets

Two datasets were explored. The first is related to cancer classification of leukemia: myeloid leukemia (AML) and acute lymphoblastic leukemia (ALL). The data comes from Golub et al. [23] and consists of two datasets of 38 (27 ALL + 11 AML) training and 34 (20 ALL + 14 AML) test samples. Each sample is associated with an individual who is encoded in a fingerprint-like fashion by means of a string of 7129 factors and a maximum of 3 levels each. The second set relies on prostate cancer data of Singh et al. [33]. The original database consisted of 1.26 × 10^4^ gene labels (at three levels each) of 102 individuals, 52 of them presenting a tumor. After discarding redundant or constant gene sets, two subsets of descriptors were obtained. Subset 1 consisted of 8053 genes presenting three levels (A, M, P), and subset 2 consisted of 2541 genes presenting two levels (M labels were set to P).

## 3. Results and Discussion

Two application examples are presented in this section. The main objective is twofold. First, it is wanted to test SSIR as a systematic way to rank cases described by symbolic general binary or multilevel fingerprints. Second, the consensus models obtained will be tested to rank gene descriptions of individuals according to distinct cancer issues. By fingerprints, we mean labels that encode gene expressions. As it will be seen, these labels define two or three levels per descriptor.

### 3.1. Gene Expression and Anticancer Activity

In 1999, Golub et al. [23] published a seminal article showing how cancer types can be classified or assigned by monitoring gene expression. The authors presented a computational algorithm able to automatically establish a distinction between AML and ALL without previous knowledge of these classes.

**Figure 2:**
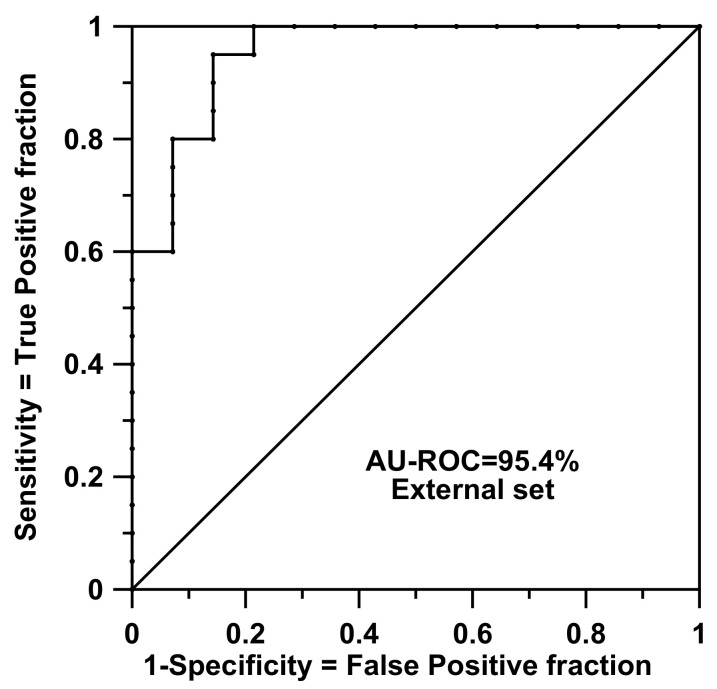
Receiver Operating Characteristic (ROC) curve for the external set of Golub and coworkers: rules are of order 2.

In order to mine the data with SSIR, the training set was inspected and all constant factors showing a single level were removed. In this way, 3645 genes of two levels (the original level M was set to A) were maintained and the calculation with the threshold *p* = 10^−7^ for rules of order 2 gave 1077 significant rules among a total of 2.6564760 × 10^7^ possible. For the training set, the fit was notable (AU-ROC = 1.0). This calculation involves generation of all rules, *p*-value calculation, selection, and application of the selected ones to the training items. The whole procedure of model building took 30 min (afterwards, the evaluation of the test items is almost immediate) in an Intel Core i7-8700@3.20GHz CPU computer with 16 GB of RAM running under 64-bit Windows 10 Enterprise. A more realistic model and procedure performance value is the one provided by the L1O cross-validation test, which decreased to AU-ROC = 0.785. Our protocol for L1O calculations is highly demanding: it involves the full repetition (from scratch) of a training calculation but, each time, leaves out one individual. At the end of each calculation, the left-out case is evaluated. However, this procedure does not scale directly according to the above 30-min timing because, when performing the training, the information related to the rules are kept in memory or a disk for later use [27,28,29]. In doing so, it is quite fast to simulate inclusions or exclusions of individuals when the rules need to be reevaluated during the L1O protocol. All in all, the whole L1O process needs about 160 s per case. As said, the application of rules on the external test set (i.e., the application of the rules for real predictive purposes) is almost immediate after training and reproduced a good result of AU-ROC = 0.954 (accuracy = concordance = 91.2%, sensitivity = 100.0%, specificity = 78.6%, precision = 87.0%, and Matthews Correlation Coefficient (CC) = 82.7%). Figure 2 shows the ROC curve prediction for this external set. This result was surprising because the training set had only 38 items and a dimensionality problem was expected to occur. The performance seemed to be independent of the fact that the test samples were collected from different reference laboratories and included a wide clinical range of samples, as Golub and coworkers stated.

An a priori reduction of the number of genes was also forced by picking up 158 genes, presenting each one almost at the same number of levels of each kind along all individuals (that is, seeking for the maximum entropy in preselected variables). Calculations were run in order to see if satisfactory results could be also obtained from this reduced set of descriptors. For this case, the cutoff was set at *p_c_* = 0.001, and 115 rules or order 2 were found. The L1O calculation returned AU-ROC = 0.939, and for the external test set, AU-ROC = 0.900 was obtained (accuracy or concordance = 85.3%, sensitivity = 75.0%, specificity = 100%, precision = 100%, and Matthews CC = 74.3%).

The results point us to the idea that it seems possible to drastically reduce the number of selected descriptor genes. Future work must be addressed to reach conclusions about the nature of the information contained in the genes used here as descriptors in models (especially those in the reduced set of 158). It is expected that rules provided by SSIR can be helpful in locating crucial gene combinations directly related to leukemia subtypes.

**Figure 3:**
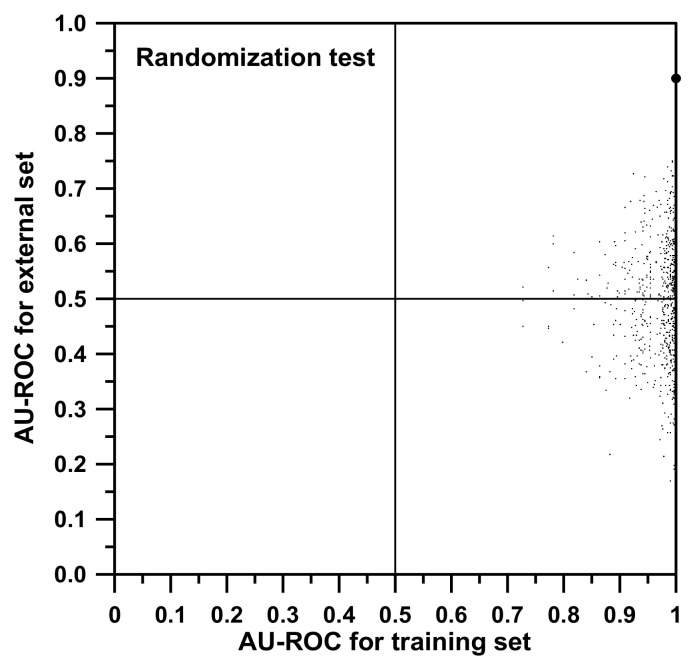
Results of the randomization test for the external set of Golub and coworkers: The reduced set of descriptors was considered, the rules are of order 2, and *p_c_* = 0.001.

Additionally, a randomization test was performed with the idea to strengthen our conclusions. In Figure 3, two axes associated with model qualifying parameters (in our cases, AU-ROC values) are represented. Then, a particular point is represented: the “true point”, the large dot at the top right of the graph which is associated with the correct model (AU-ROC values of 1.0 for training and 0.9 for the external set evaluation). After that, by randomly merging the labels of the dichotomic property studied, the data are simulated to be corrupted. In this case, this means that, among the training individuals (and not for the external set ones), the classification of types AML and ALL were randomly shuffled. Each time a shuffle is done, both the training model and the external set classification using this new model are redone from scratch (all training SSIR rules are reevaluated), obtaining a “false model”. The values of AU-ROC of the false model are expected to be worse than those corresponding to the “true model”, and this “shuffled” point is represented in the graph. A total of 1000 shuffles were performed, corresponding to the 1000 points depicted in Figure 3. A good randomization test is passed when all false models worsen the true value. After the iterations, no AU-ROC value was greater than our true reference of 0.900 for the external set.

During the shuffles, many training models reached AU-ROC values near unity. This is so for two reasons. First, the scrambling process leaves the sample labels not fully exchanged, meaning that many training items still bear correct classification to the AML or ALL cases. Second, during the training selection of rules, it is possible to overfit the model or, in other words, to generate fake models adapted to the actual scrambled training property values. However, an apparently good training result does not imply good model performance when facing (for prediction purposes) the external test set. That is the reason why models should always be verified against a validation or external set that maintains the original correct binary property classification. As it can be seen in Figure 3, despite the fact that many of the randomized training AU-ROC values are artificially high (many points present a value for training near 1.0), those of the attached external set prediction are bad. The maximum value reached for the randomized external set was 0.823. The series of collected values along the vertical axis followed a Gaussian distribution with a mean of 0.493 and a standard deviation of 0.101. This allows estimating a significant *p*-value for the true point with respect to the random distribution. The true value of 0.900 is four sigmas beyond the mean (i.e., *z* = 4.03 and *p* < 0.00003).

Along the vertical axis, it was also expected to obtain a symmetric distribution centered on the value of about 0.5 (actual value 0.493). It should be remembered that a value of 0.5 for the AU-ROC is associated with a model that discriminates in a totally random manner. In other words, it is not better than playing heads and tails. Additionally, negative AU-ROC values are associated with models that rank the samples set in the reverse order that it should be. In those cases, the model should be reversed in order to point to the correct ranking. Figure 3 shows how the “best” inverted model gives an AU-ROC of approximately 0.2. Once reversed, this is equivalent to a “correct” model that presents an AU-ROC of 0.8, almost the same value as the “best” random model.

### 3.2. Gene Expression and Prostate Cancer

Another example retrieved from the literature is that of Singh et al. [33]. Assuming that there is a link between clinical behavior of prostate cancer and the underlying differences in gene expression, these authors were able to predict patient outcome following prostatectomy. In this study, the raw data was retrieved from the supplementary information site provided by the authors. Then, a systematic application of the SSIR method showed how relevant information can be retrieved from rules of order 1 or 2, that is, when the individual rules involve two genes at most. For the two subsets, a partial optimization procedure was followed, looking at the ranking performances, while the threshold values (*p_c_*) were changed in powers of ten.

For subset 1, a total of 2.0349 × 10^4^ rules of order 1 can be generated. The best AU-ROC was found when considering only the rules that improve a value of *p_c_* = 10^−5^ attached to the L1O calculation (see Table 1). For a calculation of this kind, the training and L1O footage only took seconds because the number of rules of order 1 is small and, additionally, only a few rules were selected as significant. Reducing the cutoff of the *p*-value is expected to improve the results, since the selected rules are more restrictive and probabilistically (difficulty) demanding. On the other hand, by decreasing the value of *p_c_*, the number of selected rules also decreases dramatically, making the model more spurious or fluctuant. For *p_c_* = 10^−5^, there are still 27 significant consensus rules associated with the overall fit (see Table 1) which can make up a good cross-validation model. When the parameter *p_c_* is set to 10^−6^, only 8 rules survive and the model obtained worsens.

At the optimal point (*p_c_* = 10^−5^), the number of correct classifications is maximum. Repeating the calculations for set 2 (see Table 2), the best AU-ROC was again found for *p_c_* = 10^−5^ for the L1O test, but only 7 rules could be defined. The model obtained for *p_c_* = 10^−6^ is spurious, since only 3 training rules are defined. In fact, the training model for *p_c_* = 10^−7^ selects the same rules, but when performing the L1O procedure, both calculations select a distinct set of rule sets. That is the reason why the values of L1O AU-ROC are different.

The set of the best seven rules are those with indicators number 481, 937, 866, 2385, 1164, 52, and 315 at level *A*. All these rules have a negative vote, meaning that the presence of such a level in these indicators favors the class of nontumor state.

For subset 2, there are a total of 1.290828 × 10^7^ rules of order 2 definable. An almost optimal AU-ROC value was also found for the calculation of L1O for *p_c_* = 10^−11^, 0.923, which is obtained from a model involving the 10 most significant rules (see Table 3). This calculation also took about 30 min for training (the L1O needs less than 3 min to obtain a left-out simulation per individual). This ranking gives accuracy = 89.2%, sensitivity = 86.5%, specificity = 92.0%, precision = 91.8%, and Matthews CC = 78.6%.

For the two studied cases, the number of rules of order 2 was always exhaustively generated. In other applications (and especially if rules of higher order are to be evaluated), complete generation is not feasible due to the combinatorial explosion. In those cases, a sort of Monte Carlo (random) selection of rules can be considered. In any case (exhaustive-combinatorial or incomplete stochastic selection), training timings strongly depend on and are directly proportional to the number of definable rules. The total number of rules (of order 2) inspected for the two case studies presented is of the order of 10–25 million, and as said, the training time needed is 30 min. This can be considered competitive timing because, in cases where a top of about 500 million rules are to be generated, the timing will not reach half a day.

## 4. Conclusions

It has been shown how the SSIR procedure is useful for ranking a series of gene expressions described by categorical levels (binary descriptors or fingerprints presenting three possible levels). The mathematical foundations of the algorithm have been revisited and applied to fingerprints that encode patient transcription data to diagnose two types of cancer. Two datasets, related to leukemia and prostate cancer, have been explored to reveal the main characteristics and performance of the method. The successful results obtained are promising as the procedure not only ranks the individuals (AU-ROC values: 0.95 for leukemia case external set prediction and 0.90 for L1O for one prostate cancer subset) but also selects specific gene expressions that presumably are directly responsible of key effects. This points us to the idea of also applying the methodology to other types of diseases or affectations and case descriptors. The training time scales according to the number of rules inspected, and it only takes half an hour to inspect up to 25 million.

## Figures and Tables

**Table 1:** AU-ROC values for several calculations for subset 1: the threshold *p*-value was modified to see the variation in AU-ROC value when considering rules of order 1 for overall fit and the cross-validation leave-one-out (L1O) test. See the text for more details.

*p_c_* Value	Rules Selected	Overall Fit	L1O Performance
10^−2^	1236	0.812	0.635
10^−3^	274	0.880	0.743
10^−4^	66	0.924	0.793
10^−5^	27	0.932	0.855
10^−6^	8	0.875	0.822
10^−7^	5	0.862	0.824

**Table 2:** AU-ROC values for various calculations for subset 2: the threshold *p*-value was modified to see the variation in AU-ROC value when considering rules of order 1 for overall fit and the cross-validation leave-one-out (L1O) test. See the text for more details.

*p_c_* Value	Rules Selected	Overall Fit	L1O Performance
10^−2^	262	0.781	0.637
10^−3^	59	0.840	0.718
10^−4^	20	0.899	0.734
10^−5^	7	0.875	0.804
10^−6^	3	0.860	0.801
10^−7^	3	0.860	0.750

**Table 3:** AU-ROC values for several calculations for subset 2: the threshold *p*-value was modified to see the variation in AU-ROC value when considering rules of order 2 for overall fit and the cross-validation leave-one-out (L1O) test. See the text for more details.

*p_c_* Value	Rules Selected	Training Fit	L1O Performance
10^−3^	176,608	0.900	0.743
10^−4^	43,193	0.936	0.800
10^−5^	13,276	0.945	0.854
10^−6^	4712	0.948	0.872
10^−7^	1501	0.947	0.854
10^−8^	459	0.960	0.838
10^−9^	88	0.955	0.861
10^−10^	21	0.962	0.872
10^−11^	10	0.946	0.923
10^−12^	4	0.922	0.795
10^−13^	1	0.864	0.803
10^−14^	1	0.864	0.470
